# Preventable lifestyle and eating habits associated with gastric adenocarcinoma: A case-control study

**DOI:** 10.7150/jca.39023

**Published:** 2020-01-01

**Authors:** Lei Huang, Lei Chen, Zhong-Xuan Gui, Shun Liu, Zhi-Jian Wei, A-Man Xu

**Affiliations:** 1 Department of General Surgery, the First Affiliated Hospital of Anhui Medical University;; 2 Second Clinical Medicine College of Anhui Medical University.

**Keywords:** Gastric adenocarcinoma, lifestyle, eating habits, etiology, case-control study.

## Abstract

**Background:** Besides the well-established risk factors for gastric adenocarcinoma (GaC), many other etiological factors remain largely unexplored. This large comprehensive case-control study aimed to investigate the preventable lifestyle and eating habits associated with GaC.

**Methods:** Consecutive patients with primary microscopically-confirmed GaC diagnosed in 2016-2018 were matched by sex, age, height, and socioeconomic status at a 1:1 ratio with healthy controls. Association of GaC versus control with investigated factors was assessed using the multivariable-adjusted conditional logistic regression for paired samples.

**Results:** Together 302 GaC patients and 302 healthy controls were investigated. Participants receiving higher education and those eating majorly vegetables had less frequently GaC. The majorly frying cooking habit was associated with a higher incidence of GaC. People complaining about poor sleep quality had more often GaC. The more often one smoked, the more often he/she had GaC. A higher frequency for having pickled food was associated with more frequent GaC, while having more frequently vegetables/fruit, beans, or kelps was associated with less often GaC. A greater preference for sour or bitter taste was associated with less frequent GaC. The frequencies of thin liquid intake after meal, swallowing hot food without adequate cooling, doing other things while eating, eating overnight food, and eating midnight snack were all positively associated with GaC, while going to bed regularly was associated with less often GaC.

**Conclusions:** Education level, sleep quality, smoking, the frequencies of use of several foods and seasonings, the preference for specific tastes, and various eating and living habits were associated with GaC. The findings offer important hints for further prospective investigations and for easy effective GaC-preventative strategy-making.

## Introduction

Gastric cancer, the majority of which is gastric adenocarcinoma (GaC), is the 5^th^ most commonly diagnosed malignancy and the 3^rd^ leading cause of cancer mortality in both sexes combined worldwide, with ~1,034,000 new cases and ~783,000 deaths in 2018 1. Its incidence is highest in Eastern Asia 2. In China, gastric cancer was estimated to affect ~679,000 patients and to cause 498,000 deaths in 2015, and was both the 2^nd^ most commonly diagnosed cancer and the 2^nd^ leading cause of cancer death in both sexes combined 3.

*Helicobacter pylori* (*Hp*) is the major risk factor for GaC, contributing to ~90% of new cases of non-cardia GaC 4, 5. Some unhealthy dietary habits (*e.g.*, food preservation by salting and low fruit/vegetable intake), alcohol consumption, and tobacco smoking have also been shown to be associated with a higher risk of GaC 1, 6-13. Notably, results on the associations of GaC with some factors (*e.g.*, drinking, smoking, and red meat intake) remain controversial 1, 6-16, and many other preventable risk factors have not yet been well established.

This study aimed to comprehensively investigate the easily-modifiable lifestyle and eating habits associated with GaC and to offer evidence for disease prevention. Our findings can potentially aid to identify people at high risk of GaC and be used for risk-adapted screening.

## Methods

### Participants

Consecutive patients with first primary microscopically-confirmed GaC diagnosed in the First Affiliated Hospital of Anhui Medical University (FAHAMU) between July 2016 and August 2018 were included in this case-control study. Patients with previous malignancies, with cancers other than GaC, with other benign gastric diseases, with diseases impairing memory (*e.g.*, dementia), with severe dysfunction of important organs, or with severe systematic unfitness were excluded. They were matched by sex, age, height, and socioeconomic status at a 1:1 ratio with healthy controls confirmed not to have any gastric disorders except superficial gastritis. Since many patients with GaC are diagnosed at an advanced stage and are usually significantly thinner compared to the healthy controls and their pre-disease conditions, weight was not included as a matching factor. All participants did not have previous symptomatic reflux, and had fridges for food preservation. Individuals with any first-degree relative having GaC were excluded. Informed consents were obtained from all participants. This study was approved by the Internal Review Board of FAHAMU.

### Collected information

Participants were requested to carefully respond to a valid, uniform, and standardized questionnaire and to report their regular, habitual, customary, long-lasting conditions (before having obvious digestive symptoms in GaC patients). To ensure the validity and completeness of the responses, the completion of each questionnaire was supervised by one of the trained authors, who only explained items neutrally when necessary but did not offer any directive or indicative clues.

Information on participant characteristics (sex, age, height, weight, education level, marital status, alcohol drinking, smoking, and passive smoking) and comorbidities (hepatitis, diabetes, hypertension, and allergy) were first collected. Tumor location and differentiation were retrieved for patients. All participants were further requested to report the following: Number of people living and eating together with; eating and cooking habits; drinking water source; frequency score (FS) for intake of pork, chicken, beef, fish, processed meat, pickled food, dried food, smoked/baked food, vegetables/fruit, beans, stewed food, fried food, cereals, tuber crops, kelps, and dairy products; FS for use of yellow rice wine, soy sauce, vinegar, monosodium glutamate, chicken essence, onion/garlic, pepper, and ginger; FS for intake of thick (*e.g.*, thick soup and milk) and thin liquid (*e.g.*, water and juice) before, during, and after meal; FS for several eating habits (swallowing hot food without adequate cooling, not sufficiently chewing, overeating, doing other things while eating, eating deteriorated food, eating overnight food, eating within 0.5 hours after sports, eating midnight snack, and having milk before sleep); FS for eating at home, eating at canteen, and eating box lunch; FS for several sleeping habits (going to bed regularly, dreaming, and afternoon nap); FS for and time of housework and exercise per day; preference score (PS) for sour, sweet, bitter, spicy, and salty tastes; regularity score (RS) for having breakfast, lunch, and supper; degree of satiety and food intake per meal; rest hours after meal; nighttime and noontime sleeping hours; and quality of sleep.

FS was defined as: 0, never; 1, ≤1 time per month; 2, 2-3 times per month; 3, 1-2 times per week; 4, 3-4 times per week; 5, 5-6 times per week; 6, 1 time per day; 7, 2 times per day; 8, 3 times per day; 9, ≥4 times per day. The frequency was modified from the Food Frequency Questionnaire 17. PS ranged from 1 (extremely dislike) to 7 (extremely like) with an increment of 1. RS ranged from 1 (very regular) to 5 (very irregular) with an increment of 1.

### Statistical analyses

The paired *t* and *χ^2^* tests were used for comparing continuous and categorical variables between groups, respectively. Associations of GaC versus control with the investigated factors were first computed in basic models using the multivariable conditional logistic regression for paired samples adjusting for sex, age, and height, and the significant factors were then all incorporated into a final multivariable logistic model also adjusting for sex, age, and height. Subgroup analyses were further performed for cardia and non-cardia cancers, respectively. Statistical significance was defined by 2-sided *P*<0.05. Data analyses were performed using R 3.5.1 (https://www.r-project.org/).

## Results

### Participant characteristics

Initially 628 questionnaires were collected. After excluding the unqualified ones and their pairs, finally 604 cases (n_Patients_=302; n_Controls_=302) were analyzed. Male proportions in both groups were 69%. The mean ages in the patient and control groups were 60 ± 11 and 59 ± 11 years, respectively. Mean heights were 165 ± 7 and 164 ± 7 cm for patients and controls, respectively. For patients, the proportions of tumors located at the gastric cardia, fundus/body, antrum/pylorus, and whole stomach were 46%, 23%, 29%, and 2%, respectively, and the proportions of well-differentiated, moderately-differentiated, and poorly-differentiated/undifferentiated cancers were 5%, 25%, and 70%, respectively.

### Basic models

Compared to uneducated patients, patients going to primary school (OR=0.55), middle school (OR=0.41), high school (OR=0.45), and college/university (OR=0.25) were significantly associated with less often GaC (Table 1). Alcohol drinking was significantly associated with more frequent GaC (OR=2.46), and per 1 higher FS the odds for GaC increased by 0.10. Smoking was associated with more often GaC (OR=2.26), and the odds for GaC increased by 0.17 per 1 higher FS. The FS for passive smoking was also significantly associated with GaC (OR=1.21). No significant associations of GaC with marital status, or histories of hepatitis, diabetes, hypertension, or allergy were observed.

Regarding food and liquid intake (Table 2), the FS for intake of pork (OR=0.89), beef (OR=0.84), fish (OR=0.82), and egg (OR=0.84) was significantly inversely associated with GaC. Particularly, the number of eggs eaten per day was significantly negatively associated with GaC (OR=0.74), and not eating any eggs per day was associated with a higher proportion of GaC compared to eating 1 egg per day (OR=2.17). The FS for having processed meat (OR=1.23), pickled food (OR=1.21), dried food (OR=1.26), and smoked/baked food (OR=1.19) was significantly positively associated with GaC. Having vegetables/fruit, beans, cereals, tuber crops, and kelps decreased the odds for GaC by 0.34, 0.25, 0.18, 0.17, and 0.24 per every additional PS, respectively. The FS for use of yellow rice wine (OR=0.81), soy sauce (OR=0.89), vinegar (OR=0.84), onion/garlic (OR=0.81), pepper (OR=0.85), and ginger (OR=0.79) was significantly inversely associated with GaC, while the FS for use of monosodium glutamate (OR=1.09) and chicken essence (OR=1.07) was positively associated with GaC. The PS for sour (OR=0.86), bitter (OR=0.70), and spicy tastes (OR=0.82) was significantly less frequently associated with GaC.

Concerning eating and living habits (Table 3), the number of people living or eating together with was not significantly associated with GaC. Compared to people having majorly vegetables for food, those keeping a balanced diet (OR=2.17) and having majorly meat (OR=3.77) were significantly more likely to have GaC. The majorly frying cooking habit was significantly associated with a higher possibility of GaC compared to majorly steaming/boiling (OR=2.67). Drinking well water was significantly associated with GaC compared to tap water (OR=2.36). The RS for breakfast (OR=1.54), lunch (OR=1.77), and supper (OR=1.78) was significantly positively associated with GaC, while no significant association was shown for degree of satiety. Higher FS for thin liquid intake before meal (OR=1.13), both thick (OR=1.13) and thin liquid intake during meal (OR=1.12), and thin liquid intake after meal (OR=1.19) was associated with increased odds for GaC, while higher FS for thick liquid intake after meal was associated with less frequent GaC (OR=0.89). The FS for overeating (OR=1.47), not sufficiently chewing (OR=1.16), doing other things while eating (OR=1.13), swallowing hot food without adequate cooling (OR=1.25), eating deteriorated food (OR=1.87), eating overnight food (OR=1.16), eating within 0.5 hours after sports (OR=1.13), and eating midnight snack (OR=1.54) was all significantly positively associated with GaC, while there was no significantly association between GaC and having milk before sleep. While eating at home was significantly associated with less frequent GaC (OR=0.88 per 1 FS), eating at canteen was significantly associated with more often GaC (OR=1.12 per 1 FS). The FS for eating box lunch was not significantly associated with GaC. No significant associations were observed for rest hours after meal, or nighttime or noontime sleep hours. Compared to good sleep quality, moderate (OR=1.88) and poor quality (OR=2.81) were associated with increased odds for GaC. The more often one goes to bed regularly and has afternoon nap, the decreased odds for GaC (OR=0.80 and 0.93 per 1 FS, respectively). No significant associations of GaC with housework or exercise were observed.

### Final multivariable model

In the final multivariable model (Table 4), participants receiving primary school (OR=0.27) or middle school education (OR=0.21) had significantly less often GaC compared to those uneducated. Compared to people keeping a balanced diet, those having majorly vegetables had significantly less frequently GaC (OR=0.23). The majorly frying cooking habit was significantly associated with a higher incidence of GaC compared to the majorly steaming/boiling habit (OR=10.23). Compared to people having good sleep quality, those complaining about poor sleep quality had significantly more often GaC (OR=3.18). The more often one smoked, the more often he/she had GaC (OR=1.28 per 1 FS). Higher FS for having pickled food was significantly associated with more frequent GaC (OR=1.41), while having more frequently vegetables/fruit (OR=0.60), beans (OR=0.73), or kelps (OR=0.72) was significantly associated with less often GaC. A greater PS for sour (OR=0.77) or bitter taste (OR=0.50) was significantly associated with less frequent GaC. The FS for thin liquid intake after meal (OR=1.27), swallowing hot food without adequate cooling (OR=1.21), doing other things while eating (OR=1.23), eating overnight food (OR=1.25), and eating midnight snack (OR=1.49) was all significantly positively associated with GaC, while going to bed regularly was significantly associated with less often GaC (OR=0.81 per 1 FS).

The association patterns for cardia and non-cardia cancers were mostly similar with the whole cases, only with few exceptions. For cardia cancers, The FS for having eggs was significantly associated with less often GaC (OR=0.32). The FS for eating vegetables/fruit was more strongly associated with reduced cardia carcinoma frequency (OR=0.16) compared to overall and non-cardia carcinomas. The FS for vinegar use (OR=0.65) and the PS for spicy taste (OR=0.57) were only significantly negatively associated with non-cardia cancers. More frequent pepper use was only significantly associated with less often cardia cancers (OR=0.47), and more often thin liquid intake during meal (OR=2.27), more often chewing insufficiently (OR=2.69), more frequently eating deteriorated food (OR=7.84), and more irregular supper intake (OR=6.37) were only significantly associated with more frequent cardia cancers.

## Discussion

This study comprehensively reported eating and living habits associated with GaC in a large Chinese population, offering further insights into potentially modifiable factors and providing important evidence for making GaC-preventive strategies. Furthermore, some differences in association patterns and/or strengths between cardia and non-cardia cancers were found for some factors.

We found that people receiving primary or middle school education had significantly less often GaC compared to uneducated people, which is consistent with some previous studies showing that better education was associated with reduced GaC risk 18, 19.

Better education could help to form and keep healthy eating and living habits, while well-educated people might face greater pressure in this competitive modern era. The associations for those receiving high school and college/university education were insignificant in overall patients and most subgroups, which could be partly explained by the small case numbers in these groups. People having majorly vegetables for food had significantly less frequent GaC, which is also supported by previous studies 20, 21. Furthermore, we found that more frequent vegetable/fruit intake was significantly associated with a reduced frequency of cardia cancer but not of non-cardia cancer. Previous evidence remains controversial regarding the association between red meat intake and GaC risk 16, and The Netherlands Cohort Study did not show a significant association 15. The insignificance for the habit of majorly meat intake in our study might be partly explained by the paucity of participants in that group. The majorly frying cooking habit, which could generate various carcinogenic substances in a temperature-dependent manner, was associated with a higher overall GaC incidence compared to majorly steaming/boiling. We found that poor sleep quality was significantly associated with a higher GaC incidence compared to good quality. Notably, sleep quality could be influenced by various factors like time to go to bed and psychiatric status. We further found that the frequency of going to bed regularly was significantly associated with a reduced risk of GaC especially non-cardia cancer. Our finding that smoking was associated with GaC in a dose-dependent manner was well supported by previous literature 22; however, we did not observe a significant association for alcohol drinking frequency, on which previous evidence remains controversial 14.

The frequency of pickled food intake, a well-recognized risk factor for GaC 23, was associated with increased risks of both cardia and non-cardia cancers. Interestingly, more frequent egg intake was significantly associated with a reduced risk of cardia cancer but not of non-cardia cancer. While higher frequencies of intake of beans and kelps were both significantly associated with a decreased overall GaC risk, beans intake was significantly associated with non-cardia cancer and kelps intake with cardia cancer. More often vinegar use was significantly associated with a reduced non-cardia cancer risk, and a greater preference for sour taste was significantly associated with a lower overall GaC risk. We previously reported that distal GaC was mostly associated with hypoacidity 24, and adequate acidification of inner-stomach environment might be protective against malignancy, possibly by inhibiting growth and proliferation of organisms. More frequent pepper use was associated with a lower incidence of cardia cancer, and a greater preference of spicy taste was significantly associated with a lower cardia cancer risk. However, an early study 25 reported that Chili pepper consumption was positively associated with GaC risk. The discrepancies from our findings could be possibly due to the different strains between Asia and South America. Notably, a greater preference for bitter taste was significantly associated with reduced incidences of both cardia and non-cardia cancers. The associations with various food and seasoning intake and flavor preference offer important clues for easy GaC-preventative strategy making, which should be further validated by prospective studies.

Some specific eating habits were further found to be associated with GaC risk through multivariable analysis. The more often one had thin liquid during meal, the more probably he/she had cardia adenocarcinoma, while more frequent thin liquid intake after meal was significantly associated with an increased risk of non-cardia cancer. Thin liquid intake during/after meal could dilute the gastric liquid, thus increasing the burden of stomach. Notably, we did not observe a significant association for thick liquid intake. Swallowing hot food without adequate cooling was associated with an increased GaC risk, which might be due to the damaging effect of heat to gastric mucosa. Insufficiently chewing was associated with an increased GaC risk, which could be attributed to the increased digestive burden for the stomach. Doing other things while eating could reduce the blood flow to the stomach, potentially causing the organ to be more vulnerable. The frequencies of eating deteriorated and overnight food, which might contain increased carcinogenic microorganisms and chemical compounds, were both associated with an increased GaC risk. Among 3 meals in a day, only the irregularity degree of supper was significantly associated with cardia cancer. A short interval between having supper and going to bed could induce and accelerate reflux, a risk factor for cardia cancer. Accordingly, eating midnight snack was significantly associated with an increased GaC risk. Nearly all of these potential GaC risk factors could be modifiable. If prospectively validated, GaC-preventative strategies could be accordingly made.

This case-control study was limited by its retrospective observational nature. The associations observed do not suggest causality, and should be validated in prospective cohorts. Recall bias could affect the accuracy of the results. There could be other risk factors that have not been accounted for in this study (*e.g.*, depression). *Hp* infection status was not adjusted for in this study, considering that the measure for cancer patients might not reflect the real pre-cancer status. Some originally *Hp*-infected patients may have the infection status turn negative during the development of cancer. Furthermore, it would be difficult to know the exact duration of infection which might differ largely between the patient and control groups. The case numbers in some subgroups were not large enough to obtain statistical significance, and larger relevant studies are encouraged. Notably, the risk factors for GaC in Western people could be different from those in Asian people. Molecular and genetic risk factors could potentially further help to identify people at risk.

Our study is a large comprehensive investigation on various easily modifiable factors potentially causing GaC in Asian people. Further subgroup analyses according to tumor location were conducted. While some identified GaC-associated factors have been reported previously, there are various newly detected modifiable and preventable eating and living habits, which provide important informative clues for future investigations and which will contribute greatly to GaC prevention if validated prospectively. Once validated, the findings can serve as references for making effective population-based strategies to prevent GaC. Through health education campaigns to raise the public awareness of the modifiable and preventable factors associated with GaC, it can be expected that a significant proportion of GaC cases can be avoided in a cost-effective manner, especially for individuals without *Hp* infection who may have poorer prognosis if developing GaC 26. Our evidence-based findings provide novel clues to help to identify people at a high risk of GaC which can be potentially used for risk-adapted screening and which may contribute to early diagnosis. Modifying the validated factors may even help to prolong survival and improve quality of life for patients with GaC, and further studies in these aspects are needed.

In conclusion, education level, sleep quality, smoking, the frequencies of use of several foods and seasonings, the preference for specific tastes, and various eating and living habits were significantly associated with GaC, with some location-specific differences. Our findings offer important hints for further prospective investigations and for easy effective GaC-preventative strategy making.

## Figures and Tables

**Table 1:** Basic participant characteristics

Variable	Value/comment^1^	*Controls*	*Patients*	OR (95% CI)^2^	*P_trend_*
Education	Uneducated	64 (21)	91 (32)	1.00 (ref.)	0.001
	Primary school	84 (28)	82 (29)	0.55 (0.34-0.88)	
	Middle school	85 (29)	69 (24)	0.41 (0.25-0.67)	
	High school	32 (11)	28 (10)	0.45 (0.24-0.85)	
	College/university	33 (11)	17 (6)	0.25 (0.12-0.52)	
Marital status	Married	269 (89)	259 (87)	1.00 (ref.)	0.322
	Single	33 (11)	38 (13)	1.29 (0.78-2.15)	
Migrant	No	258 (85)	167 (62)	1.00 (ref.)	<0.001
	Yes	44 (15)	102 (38)	3.66 (2.37-5.66)	
History of hepatitis	No	296 (98)	260 (96)	1.00 (ref.)	0.310
	Yes	6 (2)	10 (4)	1.72 (0.60-4.90)	
History of diabetes	No	273 (90)	247 (94)	1.00 (ref.)	0.164
	Yes	29 (10)	16 (6)	0.63 (0.33-1.21)	
History of hypertension	No	224 (74)	212 (79)	1.00 (ref.)	0.112
	Yes	78 (26)	55 (21)	0.72 (0.48-1.08)	
History of allergy	No	293 (97)	258 (96)	1.00 (ref.)	0.260
	Yes	9 (3)	12 (4)	1.68 (0.68-4.15)	
Alcohol drinking	No	172 (57)	94 (32)	1.00 (ref.)	<0.001
	Yes	129 (43)	199 (68)	2.46 (1.70-3.57)	
	Frequency score	2 ± 3; 0 (0-3)	3 ± 3; 2 (0-5)	1.10 (1.03-1.19)	0.008
Smoking	No	106 (35)	49 (17)	1.00 (ref.)	<0.001
	Yes	195 (65)	242 (83)	2.26 (1.51-3.39)	
	Frequency score	2 ± 3; 0 (0-1)	4 ± 4; 3 (0-9)	1.17 (1.10-1.23)	<0.001
Passive smoking	Frequency score	1 ± 2; 0 (0-2)	3 ± 3; 2 (0-5)	1.21 (1.13-1.30)	<0.001

Categorical variables are shown as count (percentage [%]), and continuous variables as mean ± standard deviation. ^1^Frequency score assignment was as follows: 0, never; 1, ≤1 time per month; 2, 2-3 times per month; 3, 1-2 times per week; 4, 3-4 times per week; 5, 5-6 times per week; 6, 1 time per day; 7, 2 times per day; 8, 3 times per day; 9, ≥4 times per day. ^2^Odds ratio (OR) with 95% confidence interval (CI) for the association of each variable with gastric cancer versus control was calculated using multivariable logistic regression with adjustment for sex and age. Significant ORs are marked in bold. ref., reference.

**Table 2:** Food and liquid intake

Variable	Value/comment^1^	*Controls*	*Patients*	OR (95% CI)^2^	*P_trend_*
Pork	Frequency score	4 ± 2; 4 (3-6)	4 ± 2; 4 (3-6)	0.89 (0.81-0.98)	0.019
Chicken	Frequency score	3 ± 1; 3 (2-3)	2 ± 2; 2 (1-3)	0.95 (0.85-1.07)	0.401
Beef	Frequency score	2 ± 1; 1 (1-2)	1 ± 1; 1 (1-2)	0.84 (0.72-0.97)	0.021
Fish	Frequency score	3 ± 1; 3 (2-3)	2 ± 1; 2 (1-3)	0.82 (0.72-0.92)	0.001
Egg	Frequency score	4 ± 2; 5 (3-6)	4 ± 2; 4 (2-5)	0.84 (0.76-0.92)	<0.001
Eggs per day	As continuous	1 ± 1; 1 (1-1)	1 ± 1; 1 (1-1)	0.74 (0.55-0.98)	0.037
	0	40 (13)	65 (23)	2.17 (1.38-3.40)	0.003
	1	229 (76)	181 (63)	1.00 (ref.)	
	≥2	33 (11)	41 (14)	1.35 (0.81-2.25)	
Processed meat	Frequency score	1 ± 1; 0 (0-1)	1 ± 1; 1 (0-2)	1.23 (1.08-1.40)	0.002
Pickled food	Frequency score	3 ± 2; 2 (1-5)	4 ± 2; 4 (2-6)	1.21 (1.12-1.31)	<0.001
Dried food	Frequency score	1 ± 1; 1 (0-2)	2 ± 2; 2 (1-3)	1.26 (1.12-1.41)	<0.001
Smoked/baked food	Frequency score	1 ± 1; 0 (0-1)	1 ± 1; 0 (0-1)	1.19 (1.02-1.38)	0.028
Vegetables and fruit	Frequency score	6 ± 1; 6 (6-7)	5 ± 2; 6 (4-7)	0.66 (0.59-0.73)	<0.001
Beans	Frequency score	4 ± 2; 4 (3-5)	3 ± 2; 3 (2-4)	0.75 (0.67-0.84)	<0.001
Stewed food	Frequency score	2 ± 1; 1 (1-2)	2 ± 1; 1 (1-2)	1.03 (0.91-1.18)	0.638
Fried food	Frequency score	1 ± 1; 1 (1-2)	2 ± 1; 1 (1-2)	1.07 (0.95-1.21)	0.265
Cereals	Frequency score	3 ± 2; 3 (2-4)	2 ± 2; 2 (1-3)	0.82 (0.75-0.91)	<0.001
Tuber crops	Frequency score	3 ± 2; 3 (2-4)	3 ± 2; 3 (2-3)	0.83 (0.75-0.93)	0.001
Kelps	Frequency score	2 ± 1; 2 (1-3)	2 ± 1; 2 (1-2)	0.76 (0.67-0.86)	<0.001
Yellow rice wine	Frequency score	4 ± 2; 4 (2-6)	3 ± 2; 2 (0-4)	0.81 (0.75-0.87)	<0.001
Soy sauce	Frequency score	5 ± 2; 6 (4-6)	5 ± 2; 5 (4-6)	0.89 (0.81-0.97)	0.009
Vinegar	Frequency score	4 ± 2; 5 (3-6)	3 ± 2; 3 (2-6)	0.84 (0.78-0.91)	<0.001
Monosodium glutamate	Frequency score	3 ± 3; 1 (0-6)	3 ± 3; 3 (0-6)	1.09 (1.02-1.16)	0.006
Chicken essence	Frequency score	3 ± 3; 2 (0-6)	3 ± 3; 4 (0-6)	1.07 (1.01-1.14)	0.033
Onion and garlic	Frequency score	6 ± 2; 6 (5-6)	5 ± 2; 5 (3-7)	0.81 (0.74-0.89)	<0.001
Pepper	Frequency score	5 ± 2; 5 (3-6)	4 ± 2; 4 (2-6)	0.85 (0.79-0.92)	<0.001
Ginger	Frequency score	5 ± 2; 6 (5-6)	5 ± 2; 5 (3-6)	0.79 (0.72-0.86)	<0.001
Dairy product	Frequency score	1 ± 2; 0 (0-3)	1 ± 2; 1 (0-2)	1.01 (0.92-1.09)	0.999
Sour taste	Preference score	3 ± 2; 2 (2-4)	3 ± 1; 2 (2-4)	0.86 (0.77-0.97)	0.010
Sweet taste	Preference score	4 ± 2; 4 (2-5)	4 ± 2; 4 (2-5)	0.91 (0.82-1.01)	0.077
Bitter taste	Preference score	3 ± 1; 2 (2-4)	2 ± 1; 2 (1-3)	0.70 (0.61-0.80)	<0.001
Spicy taste	Preference score	4 ± 2; 4 (3-5)	4 ± 2; 4 (2-5)	0.82 (0.74-0.91)	<0.001
Salty taste	Preference score	4 ± 1; 4 (4-5)	4 ± 2; 4 (3-5)	0.98 (0.88-1.10)	0.747

Categorical variables are shown as count (percentage [%]), and continuous variables as mean ± standard deviation. ^1^Frequency score assignment was as follows: 0, never; 1, ≤1 time per month; 2, 2-3 times per month; 3, 1-2 times per week; 4, 3-4 times per week; 5, 5-6 times per week; 6, 1 time per day; 7, 2 times per day; 8, 3 times per day; 9, ≥4 times per day. Preference score ranged from 1 (extremely dislike) to 7 (extremely like). ^2^Odds ratio (OR) with 95% confidence interval (CI) for the association of each variable with gastric cancer versus control was calculated using multivariable logistic regression with adjustment for sex and age. Significant ORs are marked in bold. ref., reference.

**Table 3:** Eating and living habits

Variable	Value/comment^1^	*Controls*	*Patients*	OR (95% CI)^2^	*P_trend_*
No. of people living together with	As continuous	3 ± 2; 2 (1-4)	3 ± 2; 2 (1-4)	1.10 (1.00-1.21)	0.060
No. of people eating together with	As continuous	2 ± 2; 2 (1-4)	3 ± 2; 2 (1-4)	1.05 (0.96-1.15)	0.295
Eating habit	Majorly vegetables	139 (46)	70 (25)	1.00 (ref.)	<0.001
	Balanced	142 (47)	165 (59)	2.17 (1.49-3.15)	
	Majorly meat	19 (6)	43 (15)	3.77 (2.00-7.10)	
Cooking habit	Majorly steaming/boiling	280 (93)	241 (83)	1.00 (ref.)	<0.001
	Majorly frying	22 (7)	51 (17)	2.67 (1.55-4.59)	
Drinking water	Well water	47 (16)	88 (30)	2.36 (1.57-3.55)	<0.001
	Tap water	253 (84)	201 (70)	1.00 (ref.)	
Breakfast	Regularity score	2 ± 1; 1 (1-1)	2 ± 1; 2 (1-3)	1.54 (1.33-1.79)	<0.001
Lunch	Regularity score	1 ± 1; 1 (1-1)	2 ± 1; 2 (1-3)	1.77 (1.48-2.11)	<0.001
Supper	Regularity score	1 ± 1; 1 (1-1)	2 ± 1; 2 (1-3)	1.78 (1.50-2.12)	<0.001
Degree of satiety	As continuous	8 ± 4	8 ± 2	0.99 (0.93-1.06)	0.800
Food intake	On diet	42 (14)	30 (11)	0.78 (0.46-1.32)	0.593
	Normal	195 (66)	182 (68)	1.00 (ref.)	
	Overeating	59 (20)	56 (21)	1.05 (0.69-1.61)	
Overeating	Frequency score	0 ± 1; 0 (0-1)	1 ± 2; 0 (0-2)	1.47 (1.28-1.68)	<0.001
Thick liquid intake before meal	Frequency score	1 ± 2; 0 (0-1)	1 ± 2; 0 (0-2)	1.05 (0.95-1.15)	0.342
Thin liquid intake before meal	Frequency score	1 ± 2; 0 (0-1)	2 ± 3; 1 (0-3)	1.13 (1.05-1.21)	0.001
Thick liquid intake during meal	Frequency score	1 ± 2; 0 (0-1)	2 ± 2; 1 (0-3)	1.13 (1.03-1.22)	0.006
Thin liquid intake during meal	Frequency score	1 ± 2; 0 (0-1)	2 ± 2; 1 (0-3)	1.12 (1.04-1.21)	0.004
Thick liquid intake after meal	Frequency score	2 ± 3; 1 (0-5)	2 ± 2; 1 (0-3)	0.89 (0.83-0.96)	0.002
Thin liquid intake after meal	Frequency score	1 ± 3; 0 (0-2)	3 ± 3; 2 (0-5)	1.19 (1.11-1.27)	<0.001
Not sufficiently chewing	Frequency score	2 ± 2; 1 (0-4)	3 ± 3; 2 (0-5)	1.16 (1.08-1.23)	<0.001
Doing other things while eating	Frequency score	1 ± 2; 0 (0-1)	2 ± 2; 0 (0-2)	1.13 (1.04-1.22)	0.005
Swallowing hot food without adequate cooling	Frequency score	1 ± 2; 0 (0-2)	3 ± 3; 2 (0-5)	1.25 (1.16-1.35)	<0.001
Eating deteriorated food	Frequency score	0 ± 1; 0 (0-0)	1 ± 1; 0 (0-1)	1.87 (1.48-2.35)	<0.001
Eating overnight food	Frequency score	2 ± 2; 2 (0-4)	3 ± 3; 3 (1-5)	1.16 (1.06-1.26)	0.001
Eating within 0.5 h after sports	Frequency score	1 ± 2; 0 (0-2)	2 ± 2; 1 (0-3)	1.13 (1.03-1.23)	0.008
Rest hours after meal	As continuous	1 ± 1; 1 (0-1)	1 ± 1; 1 (1-1)	1.05 (0.82-1.34)	0.723
Eating midnight snack	Frequency score	0 ± 1; 0 (0-0)	1 ± 2; 0 (0-1)	1.54 (1.29-1.83)	<0.001
Eating at home	Frequency score	7 ± 2; 8 (7-8)	7 ± 2; 8 (6-8)	0.88 (0.81-0.96)	0.004
Eating at canteen	Frequency score	1 ± 2; 0 (0-0)	1 ± 2; 0 (0-1)	1.12 (1.02-1.22)	0.017
Eating box lunch	Frequency score	1 ± 2; 0 (0-1)	1 ± 2; 1 (0-2)	1.06 (0.96-1.17)	0.256
Milk before sleep	Frequency score	1 ± 2; 0 (0-1)	1 ± 1; 0 (0-1)	0.93 (0.84-1.03)	0.183
Nighttime sleep hours	As continuous	8 ± 1; 8 (7-8)	8 ± 1; 8 (7-8)	0.94 (0.84-1.06)	0.341
Noontime sleep hours	As continuous	1 ± 1; 1 (1-2)	1 ± 1; 1 (0-2)	0.99 (0.81-1.23)	0.957
Sleep quality	Good	94 (31)	46 (17)	1.00 (ref.)	<0.001
	Moderate	90 (30)	82 (31)	1.88 (1.17-3.02)	
	Poor	115 (38)	140 (52)	2.81 (1.80-4.39)	
Going to bed regularly	Frequency score	5 ± 2; 6 (4-6)	4 ± 2; 4 (1-6)	0.80 (0.74-0.87)	<0.001
Dreaming	Frequency score	3 ± 2; 3 (1-5)	3 ± 2; 3 (2-5)	1.02 (0.94-1.11)	0.608
Afternoon nap	Frequency score	4 ± 2; 4 (1-6)	3 ± 2; 4 (1-6)	0.93 (0.87-1.00)	0.048
Heavy housework	Frequency score	3 ± 3; 3 (1-6)	3 ± 3; 2 (1-6)	1.00 (0.94-1.07)	0.925
Light housework	Frequency score	5 ± 2; 5 (3-6)	4 ± 3; 5 (2-6)	1.01 (0.94-1.09)	0.724
Housework hours per day	As continuous	2 ± 2; 2 (1-4)	3 ± 2; 2 (1-3)	1.06 (0.98-1.15)	0.158
Exercise	Frequency score	3 ± 3; 3 (0-6)	3 ± 3; 3 (0-5)	0.95 (0.89-1.01)	0.081
Exercise hours per day	As continuous	2 ± 3; 1 (0-3)	2 ± 3; 1 (0-3)	1.02 (0.96-1.09)	0.491

Categorical variables are shown as count (percentage [%]), and continuous variables as mean ± standard deviation. ^1^Regularity score ranged from 1 (very regular) to 5 (very irregular). Frequency score assignment was as follows: 0, never; 1, ≤1 time per month; 2, 2-3 times per month; 3, 1-2 times per week; 4, 3-4 times per week; 5, 5-6 times per week; 6, 1 time per day; 7, 2 times per day; 8, 3 times per day; 9, ≥4 times per day. ^2^Odds ratio (OR) with 95% confidence interval (CI) for the association of each variable with gastric cancer versus control was calculated using multivariable logistic regression with adjustment for sex and age. Significant ORs are marked in bold. ref., reference.

**Table 4:** Factors associated with gastric cancer using full multivariable-adjusted model, overall and by location

Variable	Value/comment^1^	Overall gastric cancer		Cardia cancer		Non-cardia cancer	
		OR (95% CI)^2^	*P_trend_*	OR (95% CI)^2^	*P_trend_*	OR (95% CI)^2^	*P_trend_*
Education	Uneducated	1.00 (ref.)	0.014	1.00 (ref.)	0.079	1.00 (ref.)	0.012
	Primary school	0.27 (0.09-0.78)		0.03 (<0.01-0.83)		0.11 (0.02-0.64)	
	Middle school	0.21 (0.06-0.72)		<0.01 (<0.01-0.12)		0.21 (0.03-1.34)	
	High school	1.51 (0.38-6.03)		0.09 (<0.01-4.27)		1.81 (0.24-14.00)	
	College/university	0.46 (0.10-2.12)		0.20 (<0.01-15.73)		0.05 (<0.01-0.66)	
Eating habit	Majorly vegetables	0.23 (0.09-0.56)	0.003	0.03 (<0.01-0.44)	0.037	0.11 (0.02-0.46)	0.011
	Balanced	1.00 (ref.)		1.00 (ref.)		1.00 (ref.)	
	Majorly meat	0.34 (0.10-1.19)		0.32 (0.01-9.57)		0.43 (0.07-2.62)	
Cooking habit	Majorly steaming/boiling	1.00 (ref.)	0.001				
	Majorly frying	10.23 (2.70-38.80)					
Sleep quality	Good	1.00 (ref.)	0.015				
	Moderate	1.01 (0.38-2.70)					
	Poor	3.18 (1.23-8.23)					
Smoking	Frequency score	1.28 (1.12-1.46)	<0.001	1.64 (1.12-2.41)	0.012	1.32 (1.07-1.61)	0.009
Egg	Frequency score			0.32 (0.12-0.83)	0.019		
Pickled food	Frequency score	1.41 (1.16-1.71)	<0.001	2.00 (1.10-3.63)	0.023	1.55 (1.12-2.14)	0.008
Vegetables and fruit	Frequency score	0.60 (0.46-0.79)	<0.001	0.16 (0.06-0.45)	0.001	0.73 (0.51-1.05)	0.094
Beans	Frequency score	0.73 (0.55-0.97)	0.032			0.54 (0.35-0.86)	0.009
Kelps	Frequency score	0.72 (0.54-0.96)	0.026	0.24 (0.09-0.61)	0.003		
Vinegar	Frequency score					0.65 (0.46-0.92)	0.015
Pepper	Frequency score	0.84 (0.68-1.03)	0.090	0.47 (0.26-0.83)	0.010		
Sour taste	Preference score	0.77 (0.59-1.00)	0.047				
Bitter taste	Preference score	0.50 (0.36-0.69)	<0.001	0.14 (0.05-0.41)	<0.001	0.38 (0.22-0.65)	0.001
Spicy taste	Preference score					0.57 (0.38-0.86)	0.008
Thin liquid intake during meal	Frequency score			2.27 (1.12-4.61)	0.024		
Thin liquid intake after meal	Frequency score	1.27 (1.06-1.52)	0.008			1.45 (1.12-1.87)	0.005
Swallowing hot food without adequate cooling	Frequency score	1.21 (1.00-1.47)	0.045			1.44 (1.07-1.95)	0.017
Supper	Regularity score			6.37 (1.20-33.88)	0.030		
Not sufficiently chewing	Frequency score	1.18 (1.00-1.40)	0.058	2.69 (1.48-4.86)	0.001		
Doing other things while eating	Frequency score	1.23 (1.01-1.50)	0.041			1.50 (1.10-2.05)	0.011
Eating deteriorated food	Frequency score	1.37 (0.95-1.96)	0.093	7.84 (2.32-26.49)	0.001		
Eating overnight food	Frequency score	1.25 (1.03-1.52)	0.025			1.57 (1.13-2.18)	0.007
Eating midnight snack	Frequency score	1.49 (1.09-2.03)	0.013	3.57 (1.04-12.30)	0.044	2.04 (1.22-3.41)	0.007
Going to bed regularly	Frequency score	0.81 (0.68-0.96)	0.018			0.74 (0.56-0.96)	0.025

1Frequency score assignment was as follows: 0, never; 1, ≤1 time per month; 2, 2-3 times per month; 3, 1-2 times per week; 4, 3-4 times per week; 5, 5-6 times per week; 6, 1 time per day; 7, 2 times per day; 8, 3 times per day; 9, ≥4 times per day. Preference score ranged from 1 (extremely dislike) to 7 (extremely like). Regularity score ranged from 1 (very regular) to 5 (very irregular). 2Odds ratio (OR) with 95% confidence interval (CI) for the association of each variable with cancer (overall, cardia, and non-cardia) versus control was calculated using multivariable logistic regression with adjustment for sex, age, and all significant variables identified in the preliminary models adjusting for sex and age only. Results with P < 0.10 are shown, and significant ORs with P < 0.05 are marked in bold. ref., reference.
